# Advanced Control Systems in Industry 5.0 Enabling Process Mining

**DOI:** 10.3390/s22228677

**Published:** 2022-11-10

**Authors:** Alessandro Massaro

**Affiliations:** 1 LUM Enterprise S.r.l., S.S. 100-Km.18, Parco il Baricentro, 70010 Bari, Italy; massaro@lum.it or massaro@lumenterprise.it; 2 Dipartimento di Management, Finanza e Tecnologia, LUM—Libera Università Mediterranea “Giuseppe Degennaro”, S.S. 100-Km.18, Parco il Baricentro, 70010 Bari, Italy

**Keywords:** Process Mining, Industry 5.0, self-adaptive machine parameter setting, BPMN process workflows, Artificial Intelligence

## Abstract

This paper merges new research topics in Industry 5.0 using the Business Process Modeling and Notation (BPMN) approach able to integrate Artificial Intelligence (AI) in production processes. The goal is to provide an innovative approach to model production management in industry, adopting a new “proof of concept” of advanced Process Mining (PM) automatizing decisions and optimizing machine setting and maintenance interventions. Advanced electronic sensing and actuation systems, integrating supervised and unsupervised AI algorithms, are embedded in the PM model as theoretical process workflows suggested by a Decision Support System (DSS) engine enabling an intelligent decision-making procedure. The paper discusses, as examples, two theoretical models applied to specific industry sectors, such as food processing and energy production. The proposed work provides important elements of engineering management related to the digitalization of production process matching with automated control systems setting production parameters, thus enabling the self-adapting of product quality supervision and production efficiency in modern industrial systems.

## 1. Introduction

The model of process workflows is fundamental for process mapping in industry. The possibility of mapping “AS IS” and “TO BE” processes allows one to optimize production, product quality and organization management. In the new Industry 5.0 scenario, process mapping can be improved by applying Artificial Intelligence (AI) decision making algorithms mainly behaving as workflow checkpoint-defining processes: AI is able to process production data by suggesting optimized sub-processes and by defining risks related to production and product quality. The process workflow merging AI algorithms in decisional logics is named Process Mining (PM) [1,2,3]. PM constitutes a Decision Support System (DSS) engine able to predict corrective actions including machine parameter setting adjustments [1,4,5], predictive maintenance actions [1,6,7], and interventions by Predictive Process Monitoring (PPM) approaches [8]. The impact of advanced digital technologies such as AI integrated in industrial information systems [9] plays an important role in organization management [10]. In this direction, Change Management (CM) [11] models could support process re-engineering [12,13], thus optimizing production and product quality. AI is an important tool also for sensing and actuation processes, including electronic implementations supporting production and automated testing [14]. The analyzed state of the art enhances the importance of AI in the new era of Industry 5.0 improving self-adaptive production processes. The self-adaption concept suggests to apply PM to intelligent monitoring and automation. The production monitoring process can be activated by means of sensors placed in strategic parts of the production layout and detecting parameters useful for quality control. Similarly, advanced mechatronic interfaces [15] implementing AI control [1] behave as Human–Machine Interfaces (HMIs), “transforming” the information of sensors into an optimized parameter setting list of production machines. To correctly program the HMI, it is necessary to define the logic managing the monitoring and the actuation processes. The first step to design the logic of processes is to define how data processing phases are involved in the whole monitoring and control processes. Sensing and actuation functions can be primarily modeled by workflows representing the HMI functions of a specific production machine. Business Process Modeling and Notation (BPMN) is a method suitable for process workflow implementation, as well as for predictive maintenance [16] and information system applications [17]. According to electronic control and actuation processes, BPMN workflows are also useful to modeling intelligent logics based on AI [18]. The state of the art suggests we focus the attention of this study on PM model matching in the new Industry 5.0 scenario, investigating innovative workflows to design efficient schemes of production management. By following this main goal, the paper is structured into the points listed below:

- We provide a new theoretical model of PM integrating a DSS based on AI-supervised and unsupervised algorithms;
- We discuss an application field applying PM in food roasting process (application of AI unsupervised algorithm);
- We discuss an application field applying PM in energy production (application of AI supervised algorithm);
- We analyze different aspects concerning electronic/mechatronic implementations and procedures;
- We discuss PM and AI aspects as regards impacts in the supply chains and in organizational processes, thus providing an overview of different possible implementations of the proposed PM model.

The topics discussed in the paper’s sections are addressed in the context of an Industry 5.0 scenario. In Table 1 are summarized some aspects matching paper topics and Industry 4.0 and Industry 5.0 scenarios. 

## 2. Materials and Methods: Process Mining (PM) Model and AI Integration

**Figure 1:**
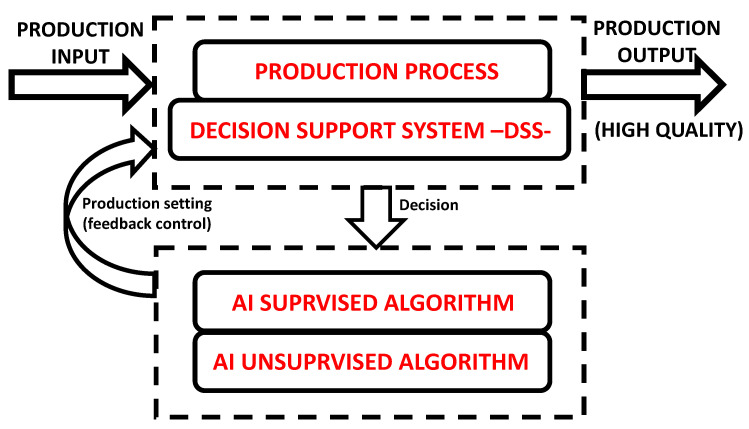
“Proof of concept” of PM.

The block diagram of Figure 1 explains the “proof of concept” of the proposed PM model based on the matching between a DSS and the standard production processes. The production input (raw materials, semi-products, elements able to produce energy, etc.) is processed by the production machine, and the related product (output) can be characterized by a high quality due to the automatic feedback control involving AI decision. The AI supervised or unsupervised algorithms are able, in cases of anomalies detection, to change in time machine parameters or to adopt interventions depending on sensor data values (values compared with thresholds indicating possible alerting conditions). 

**Figure 2:**
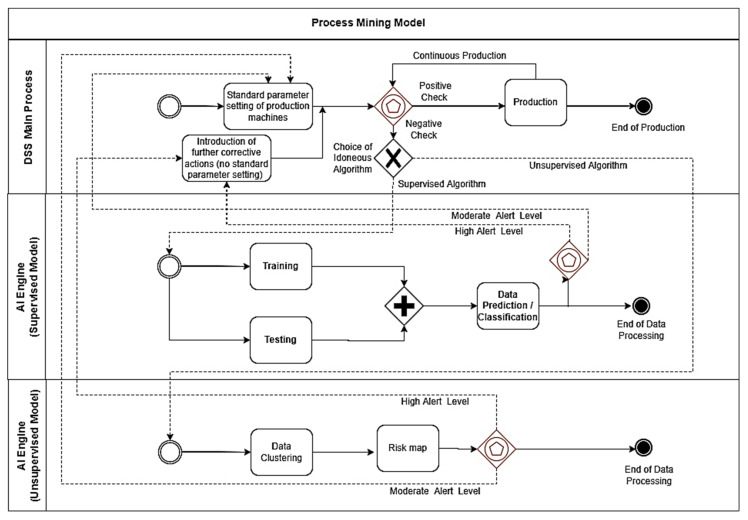
Proposed BPMN Process Mining (PM) theoretical model structured in three pools and integrating artificial intelligence algorithms (PM-AI): DSS main process and AI engine sub-processes behaving as feedback systems and providing decisions about the setting of machine parameters and about further machine corrective actions. In red are the BPMN symbols behaving as process checkpoints (“Exclusive Event based” gateways). The model indicates two alerting levels (moderate alert level and high alert level). The model can be made more complex by considering more alerting levels defining risk maps with more risk levels.

The BPMN approach is adopted to “explode” in Figure 2 the “proof of concept” of the PM theoretical model of Figure 1, and to apply the PM in the different application fields. The BPMN-PM model is designed by means of the open source Draw.io tool [19]. The BPMN notations contain some symbols, such as pools and task boxes, start and end events, exclusive and parallel gateways, and finally exclusive event-based gateway modeling process checkpoints supported by AI algorithms. AI supervised and unsupervised algorithms are implemented for the specific case studies by executing Konstanz Information Miner (KNIME) workflows [20,21] (see details in Appendix A and Appendix B about the applications discussed in this work). The theoretical approach is constructed by means of BPMN enhancing the following main functions of the PM model sketched by the three BPMN pools of Figure 2, containing the following sub-processes:

- **DSS Main Process.** This pool represents the main process of the PM model, and integrates the DSS enabling supervised and unsupervised AI algorithms. The choice of the algorithm is based on the available dataset typology. The decision to select AI data processing is established by means of the “Exclusive Event based” gateway: in the case of a positive check of the monitored variables the production process continues; besides this, in the case of negative check, the AI algorithm will be able to optimize the machine parameter setting or to decide the intervention to perform. According to the alerting level detected by the AI algorithms, a standard parameter setting (moderate alerting level requiring a soft variation of the machine parameters) or a non-standard parameter setting (high alerting level requiring a strong variation of machine parameters or further corrective interventions) will be activated. Corrective actions include predictive maintenance, possible slowdowns in production, major control by enforcing human resource operation, etc.;
- **AI Engine (Supervised Model).** This pool concerns the application of the supervised AI algorithm in processing data by means of the training and testing phases. The outputs are predicted or classified data defining alerting risk maps (two alerting levels are considered in the simplified model of Figure 2) driving parameter setting and interventions. The supervised models are preferred when a significant production variable to control is identified (labeled variable);
- **AI Engine (Unsupervised Model).** This pool represents the data clustering process defining the risk maps based on the alerting levels [22]. The output are the data clusters indicating the risk maps (two alerting levels are considered in the simplified model of Figure 2). The unsupervised models are preferred when there are more variables to control without knowing the “weight” of each variable for the specific production process.

**Figure 3:**
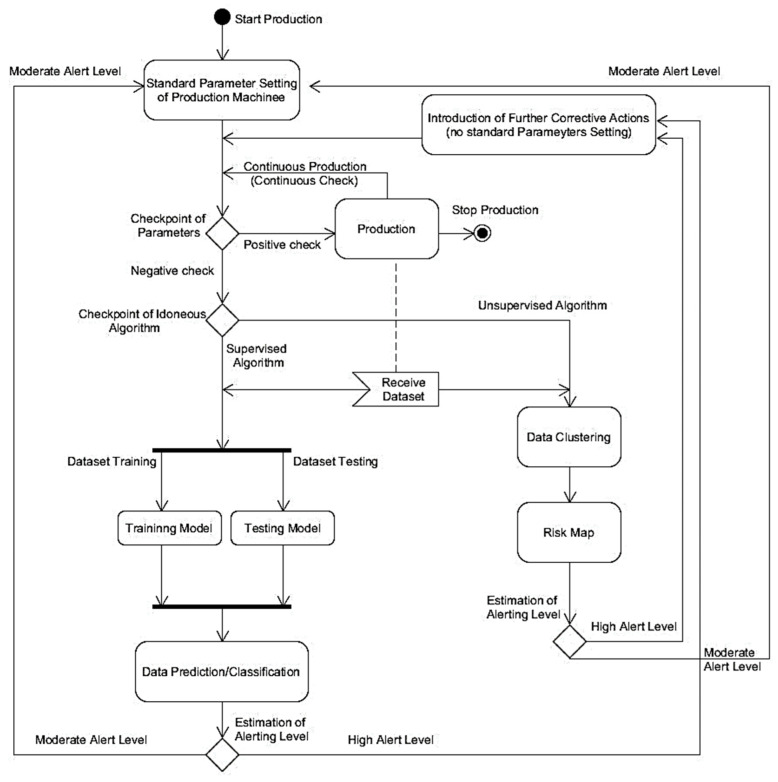
UML-AD theoretical model of the proposed PM.

The BPMN model of Figure 2 is the “translation” of the theoretical Unified Modeling Language (UML) Activity Diagram (AD) sketched in Figure 3.

The UML-AD model and the BPMN provide information about the logic to implement in the monitoring process. The logic explaining the data flow indicated in Figure 2 and Figure 3 is described by the following pseudocode (Algorithm 1):
**Algorithm 1: PM pseudocode*****Start****production process;****Initial setting of production machine;******If****check of machine is positive **Then** continue production (and parameter checking) until production ends;****Else****(negative check) choose the suitable idoneous algorithm;*   ***If** the unsupervised algorithm is **‘True’ Then** perform data clustering and*   *structure risk maps;*       ***If** a moderate alert is estimated then set the standard machine*       *parameter;*      ***Else** (high alert) introduce further corrective actions;*      ***End If;***
   ***Else (False** of the unsupervised algorithm) selection of the supervised*    *algorithm providing data prediction/classification through training and*   *testing models;*        ***If** a moderate alert is estimated then set the standard machine*        *parameters;*        ***Else** (high alert) introduce further corrective actions;*        ***End If;***
   ***End If;***
***End If.***

The DSS of Figure 2, further explained in Figure 3, is general, and can be applied, with appropriate modifications, in different application fields. The proposed work discusses the following application examples considered quite significant for understanding the PM model:

1. The roasting process of a food product passing into five ovens;
2. The energy generation process of a Combined Cycle Power Plant (CCPP).

**Figure 4:**
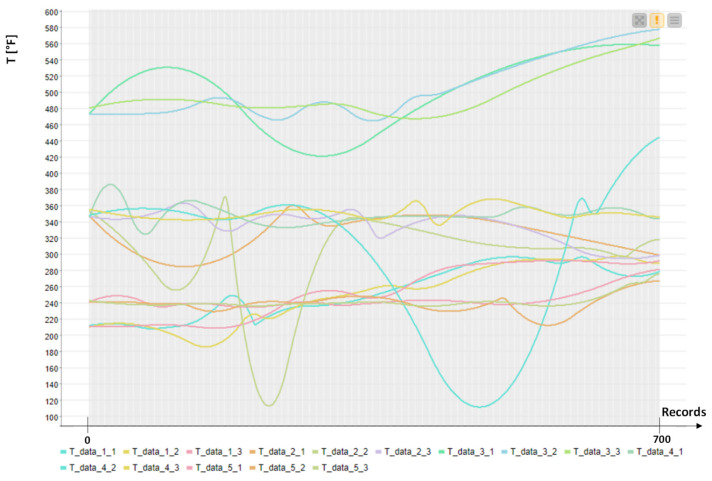
Temperature trends: first 700 records (sorted in time) extracted from the open dataset [23]. The sampling time is 1 min.

For the first application field, data from [23] are referred to for five ovens, each with three temperature sensors. In addition, we consider data about the height of the food (raw food material) and its moisture content as measurements detected when raw materials enter the machine. The application of the PM model is focused on temperatures with a major weight if compared to the other parameters. In Figure 4 are illustrated some temperature trends of all the temperature sensors (three temperature sensors for each oven). The open dataset [23] is processed for the application of the unsupervised k-Means [24] algorithm (algorithm preferred for a large number of available variables), which comprises 2,103,841 records. 

For the second application, we consider another open dataset [25] with 9568 records collected by a CCPP producing energy. The dataset refers to a time period of six years, for a power plant working in a full load condition. The CCPP plant is composed of gas turbines, steam turbines and heat-recovery steam generators. The dataset [25] is constituted by the following hourly average values: ambient Temperature (T), Ambient Pressure (AP), Relative Humidity (RH), Exhaust Vacuum (V), and electrical energy output (PE) of the plant. 

**Figure 5:**
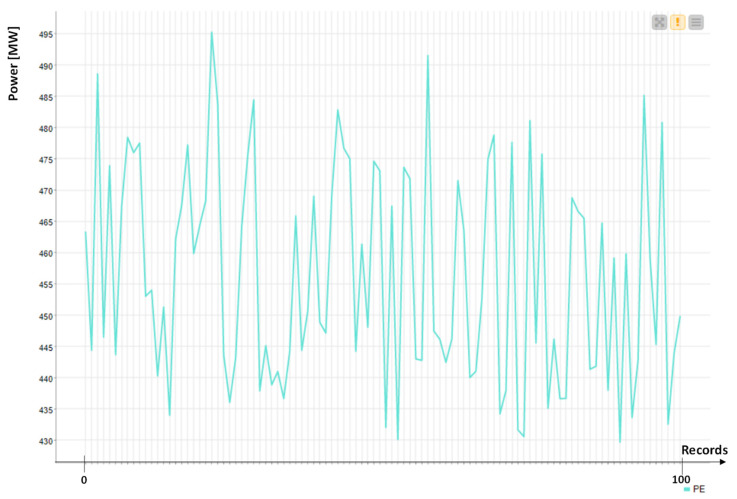
Produced energy power: first 100 records of the dataset [25].

An example of power energy trend is illustrated in Figure 5. The variable PE is the most significant, and EP is considered as the labeled variable (variable having a high “weight” as a key efficiency parameter). The AI-supervised algorithm applied for power prediction is the Artificial Neural Network (ANN) Multilayer Perceptron (MLP) algorithm typically used for electric energy prediction [26].

The method applied in this work is used to show how it is possible to use AI results following the PM model to optimize production and quality. In this direction, Section 3 provides more details about the two significant examples of PM application. 

## 3. Results: Applications of the PM Model

The PM model is applied to two processes related to food roasting and energy production. For each case, we apply the theoretical scheme of Figure 2, which is modified according to the specific processes. 

### 3.1. AI Engine (Unsupervised Model): The Roasting Process 

**Figure 6:**
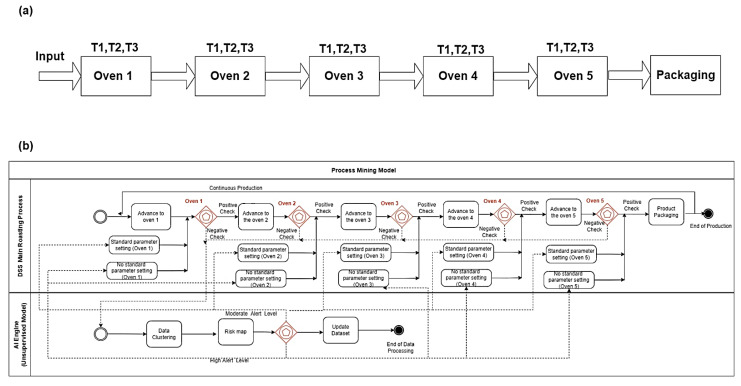
(**a**) Basic scheme of the roasting production process concerning the passage of the product into five ovens, where each oven is monitored by three temperature sensors providing parameters T1, T2 and T3. (**b**) The BPMN PM model representing the automatic setting of oven temperatures by means of an AI unsupervised engine (application of k-Means algorithm).

The first example of the application of PM is described in Figure 6a, illustrating the roasting process involving the passage of a food product into five ovens connected in series. The scheme simplifies the roasting process flux by means of a block diagram indicating the roasting process input, the passage of the product through each oven, and finally the packaging phase. Each oven is controlled by different sensors. The key parameter controlling the roasting process is the temperature. The food product passes initially into the input of the first oven, and successively into the other ovens, thus completing the roasting process. The roasted product is packaged in the final production step. Typically, the quality in the roasting process is checked by controlling temperatures [27] or by adopting advanced technologies such as Near-Infrared Spectroscopy (NIRS) [28]. A basic approach to check quality is the verification of the heat homogeneity in each oven by reading temperature values; for this, the clustering approach is indicated to check the heat stability data clusters with values confined in a limited region. Figure 6b shows the theoretical PM model applied to the roasting process as a modification of the theoretical model of Figure 2. Five “Exclusive Event based” gateways allocated in series are able to check the temperature stability in each oven. In the case of a positive check of temperature values, the oven setting remains the same, while in the case of a negative check, we perform a clustering k-Means (AI unsupervised algorithm) analysis providing a risk map with two alerting levels. The risk map can provide a moderate alert enabling the standard parameter setting of the oven, or a high level of alert requiring a stronger setting (no standard parameter setting) or a specific maintenance intervention.

**Figure 7:**
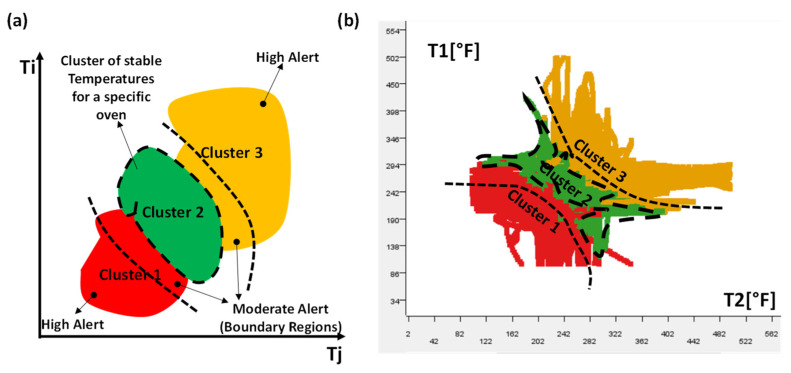
(**a**) Alerting risk map: theoretical clusters (k = 3) grouping temperature values of an oven. (**b**) Clusters and risk map estimated for the analyzed dataset [23].

The k-Means algorithm is able to provide a risk map by which it is possible to distinguish different regions. By considering for example of three clusters (k = 3), the region named cluster 2 of Figure 7a characterizes a stable condition (variation of temperature in a limited range defining the heat stability), while the other two clusters (cluster 1 and cluster 3 of Figure 7a) can be representative of an unstable condition (temperature with too high or too low values). The choice of k = 3 is a good compromise between the simplicity of analysis (few boundary regions are defined) and the performance of the clustering algorithm. In the theoretical risk map of Figure 7a, the moderate alerting level is associated with the boundary regions of cluster 3 and cluster 2 close to the stable cluster 2. A risk map is designed for the analyzed dataset of each oven (see Appendix A). In Figure 7b is illustrated an example of a risk map obtained by the k-Means clustering analysis mapping the T1 and T2 values of the first oven. Each clustering analysis is performed for each oven (for example, for oven 1, the algorithm provides clustering between T1 and T2, between T1 and T3, and between T2 and T3, and so on for the other ovens), thus achieving similar risk maps highlighting stable regions (see Appendix A). 

### 3.2. AI Engine (Supervised Model): Energy Production 

**Figure 8:**
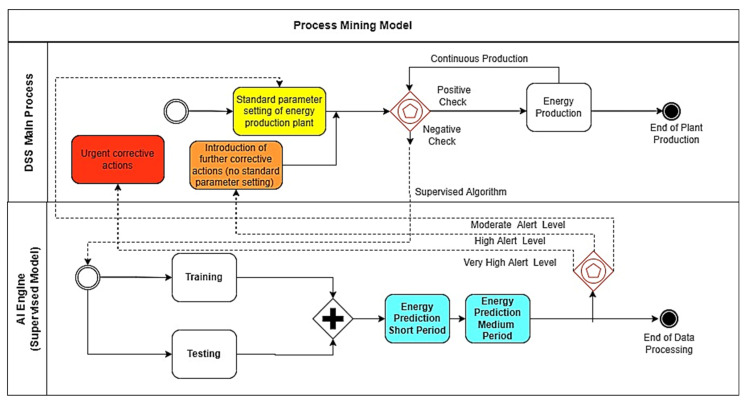
BPMN workflow modeling PM applied to CCPP plant and matching with energy power prediction.

The second example of the BPMN PM model is illustrated in Figure 8: the BPMN workflow indicates the energy production process of a CCPP plant. The AI data processing (training and testing phase) facilitates the prediction of energy in the short and medium periods. In this specific case, we executed an ANN-MLP algorithm estimating the forecasting of the energy production of the plant. The prediction results, together with the analysis of historical data, are useful to check possible power plant breakdown conditions. The PM model is able to identify three alerting level conditions: a moderate alert level (requiring a standard parameter setting of the energy production plant), a high alert level (activating further corrective actions), and a very high alerting condition (indicating urgent corrective actions to take). 

**Figure 9:**
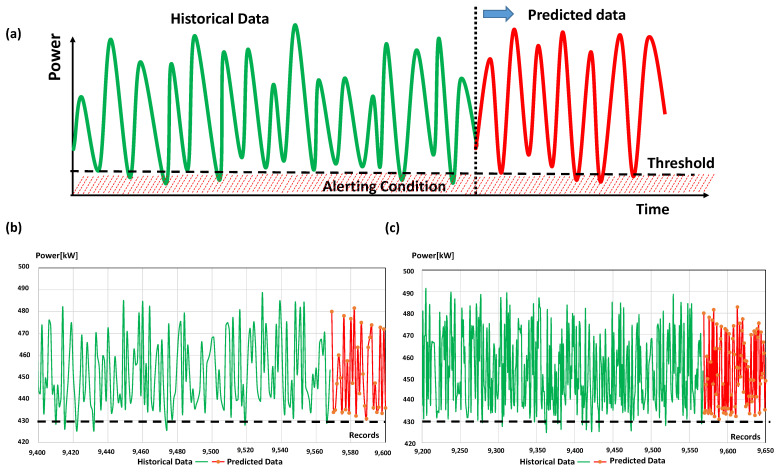
(**a**) Theoretical trend of energy distinguishing historical data (green plot) from predicted ones (red plot): the values under the threshold indicates an alerting condition (risk condition). (**b**) Historical data (green plot) [25] and ANN-MLP results (red plot) in the short period. (**c**) Historical data (green plot) [25] and ANN-MLP results (red plot) in the medium period. According to the indicated threshold, it is observed for the analyzed case a no accentuated risk condition (no alerting about production of energy power).

Figure 9a shows the theoretical trend of energy, distinguishing historical data (green plot) from those predicted (red plot).

By observing the historical data, the alerting status is identified when the energy is under a threshold value during a sufficient observation period (see horizontal dashed line of Figure 9a representing the threshold delimiting the risk region of low produced power). The observation of an alerting condition in the analyzed power trend enables the automatic prediction calculus to estimate the future alerting risk level; when the predicted results highlight a lower energy power value (compared with the threshold value) for a different period and in a continuous way, it defines a high or a very high risk level. In Figure 9b,c are illustrated the energy power trends and the power forecasting of the analyzed dataset [24] in the short and in the medium period, respectively. Each predicted sample refers to a period of about 5 h (average time estimated by considering the average time gap between two time-sorted records). The upper limit of the threshold is not defined in the analyzed case because no strange peaks are observed (for example, due to an energy overload condition), and because a high amplitude of power represents a good parameter of merit. In Appendix B are reported more details about the ANN-MLP algorithm used to predict power. 

## 4. Discussion

**Figure 10:**
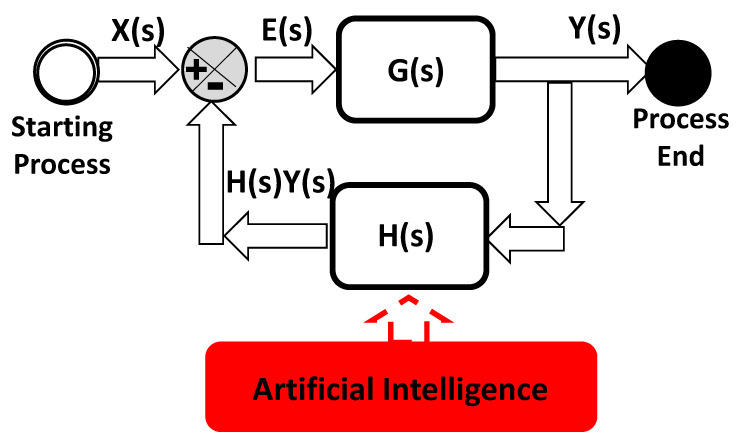
AI self-adaptive model: DSS and automatic machine parameter setting by means of AI matching with electronic feedback control and actuation systems.

A possible evolution of the PM model is its implementation in automated production systems. Specifically, AI tools can be interfaced, by means of Human–Machine Interfaces (HMIs), with production machines, providing an automated actuation control driven by the AI alerting output (through a translation via Programmable Logic Controller (PLC) protocols of the output into an executable command driving machine). In Figure 10 is illustrated the scheme of a possible matching between AI and HMI, thus controlling the error; the feedback control system is defined by *G*(s) transfer function forwarding gain, while *H*(*s*) is the feedback transfer function, and *E*(*s*) is the error signal equal to the difference between the input signal *X*(*s*) and the feedback signal *H*(*s*)*Y*(*s*).

*E*(*s*) = *X*(*s*) − *H*(*s*) · *Y*(*s*) (1)

The output signal *Y*(*s*) is provided by the equation:

*Y*(*s*) = *G*(*s*) · *E*(*s*) (2)

The error signal may be a gap measured between the desired value of the monitored parameter and the real measured one. The whole feedback system of Figure 9 is the “translation” of the PM model in terms of electronic signals, and describes a self-adaptive approach using AI as for Industry 5.0 facilities [1]. 

AI circuits based on feedback control are suitable for different industry 5.0 applications and functions, such as [1]:

- tool speed regulation integrating AI intelligent control (DC motor controlled by a voltage signal as output of the AI engine);
- tool speed regulation integrating AI controlling an electrical current traveling in series resistors;
- tool speed regulation integrating AI controlling values of series of resistors;
- tool speed regulation integrating AI controlling the value of a single resistance;
- collaborative exoskeletons with auto-adaptive solutions controlling motion trajectory and torque;
- AI controller for current-source inverter circuits (control of the switching conditions) as for three-phase Current-Source Inverters (CSIs);
- intelligent converter control;
- wave rectifier control;
- control of voltage-source inverters;
- control of current-source inverters;
- robotic PID controller based on AI learning;
- AI controller adjusting tool trajectory;
- AI-based image processing selecting inline objects (unsupervised algorithms);
- soft robotics for intelligent collaborative robotics reading disturbance measurements;
- additive manufacturing control (pulsed signal techniques);
- AI image vision circuits implementing AND logic ports.

The discussed examples will help readers to understand how the PM model can be applied in different production scenarios: as the theoretical model is flexible, it is applicable in different cases also involving substantial changes in production approaches and in organizational models. The AI analysis affects the organizational model of industries, because it activates a series of corrective interventions depending on the detected machine parameters. In this scenario, the application of CM models [11,29] is essential to achieving the best production efficiency for industries working in different sectors. 

In Table 2 are identified some aspects that are consequence of applying AI in PM models, correlated with organizational influences for different management classifications. 

In Table 3 are listed some advantages and disadvantages of the BPMN-PM models integrating AI unsupervised and supervised algorithms. 

The proposed BPMN-PM approach is mainly characterized by its limited ability to automatically define the alerting thresholds for specific cases; this requires an initial accurate analysis of historical data with the continuous checking of product quality to choose the best set of machine parameters. The challenge of future works is to implement a totally automatic system able to auto-calibrate the thresholds as a function of the quality parameters using the feedback control of Figure 10. 

## 5. Conclusions

The proposed work provides a new concept of process mapping based on AI integration into automated decision-making processes. In particular, we discuss a new concept of the PM model activating AI unsupervised and supervised algorithms and improving production processes. In order to facilitate the compression of the proposed model, we have used the BPMN approach. Our paper shows two production cases to show how it is possible to apply the theoretical model to a specific case of production. The discussed PM approach highlights other correlated aspects, including the organizational impacts, the formulation of risk maps based on the prediction of production parameters, the self-adapting processes setting machine parameters, and the possible integrations of PM with HMI. The PM model represents the first step for the implementation of advanced Industry 5.0 processes in production systems. The PM approach can be adopted to design all the advanced electronic and mechatronic systems characterized by checkpoint logics and automated parameter setting. 

## Figures and Tables

**Table 1:** Main topics of the paper and relations to Industry 4.0 and Industry 5.0 scenarios.

Topic	Industry 4.0 Scenario	Industry 5.0 Scenario [1]
PM	Industrial process are typically mapped by BPMN approach: standard workflow defines a static representation of the scenario where digital sensors transmit data and actuation systems are not managed by the same PM model.	Processes are mapped by considering decisional logics integrated in the PM model: the process is dynamic and the choice of the sub-process is a function of the real-time automatic decisions performed by a calculus unit (implementation of decision-making logics depending on AI output).
DSS	The user working on a production machine can control and manage the machine parameter through cloud applications reading sensors and enabling remote actuation. The main function of the DSS is to provide alerting signals that can be monitored online.	The DSS is a standalone system that auto-adapts the machine parameter’s setting depending on the self-learning approach: the historical sensor data are used to implement DSS detecting alerts, but also to automatically set optimal machines and tools for self-adaptive production.
Electronic components	Electronic components are mainly used for the realization of sensors and Human–Machine Interfaces (HMIs) applied on production machines.	Innovative advanced electronic systems can be manufactured by developing electronic chips and boards integrated in the production machine, and having AI logics (implementation of McCulloch–Pitts neurons by transistors and other electronic components reproducing the AI logics by logic ports). The Machine to Machine (M2M) interfaces are managed by AI networks based on feedback systems.

**Table 2:** Classes of management types and related PM-AI aspects in the supply chains and their organizational impacts.

Management Class in Industries	Supply Chain Aspects Generated by PM Models	Organizational Impact Description	AI References
Production of products	Mechatronic and electronic components interfaced via the AI algorithm providing the self-adaption of the machine parameter setting.	An accurate human resource training approach focusing on advanced electronic and mechatronic technologies is necessary for the best production efficiency. The accurate reading of AI-predicted results optimizes a possible predictive maintenance procedure or the corrective action plans avoiding defects.	[1]
Logistics	Intralogistics and logistics improvements suggested by AI-DSS (load prediction, priorities of transport activities, fuel consumption optimization, etc.).	We require a formulation of Key Performance Indicators (KPIs) oriented towards the optimization of logistics using the available resources and layouts (human resources, vehicles, etc.). CM models, together with AI results (for example, regarding load prediction), could support all the logistics activities.	[30,31]
Energy	The whole supply chain must be “energetically efficient” to reduce the high costs due to the energy consumption rate. The energy monitoring and the AI energy forecasting are important tools to reduce costs.	Energy consumption monitoring, especially for energy-intensive industries, suggests new production layouts and a possible re-organization of the whole production process deciding priorities according to the product request in the market (very high impact).	[32]
Services and micro-services	Companies working in services require AI tools to optimize marketing actions and customer care (as for recommender systems). The parameters to assess are KPI associated with the implementation of services.	We require new knowledge based on information about customer profiles and customer behavior, together with a strategic provisional analysis of marketing.	[33]
Rapid Prototyping (RP)	PM-AI models are applied for a specific phase of the production of prototypes.	PM is important to defining the best way to perform rapid prototyping (RP) in short times, thus helping the manager to decide “in time” and to continue onto the next development phase of the pre-series production. RP requires a structured team with different skills.	[34]
Human Resource (HR)	Selection processes of HR improved by executing PM-AI models.	Human capital in industries is very important for the organization and for production efficiency. CM models are matched with PM ones, thus structuring in the best way all the supply chain activities and the most suitable teams to execute specific processes.	[35,36,37]
Reverse Engineering (RE)	RE can be modeled by a PM model. This new concept of RE could optimize company strategies regarding quality process optimization.	The RE processes require advanced technologies (3D scanners, feelers, etc.) and well-defined procedures to detect object shapes with a specific tolerance depending on the accuracy of the adopted tool. Workers should be continuously trained on the updating of technologies and procedures. This requires an investment plan for the company in the training of its personnel.	[38]
Quality check	All quality processes concerning product quality checks by AI.	The quality processes drastically influence production and marketing strategies. A new concept of quality checking by means of the execution of PM models reliably optimizes the checkpoint definition (according to ISO 9001:2015 standard). The implementation of new quality processes involving AI technologies requires a revision of the organizational model related to quality control. In this area can be applied CM models closely related to quality.	[39,40,41,42]
Project Management (PM)	PM can be applied also for the management of projects regarding different sectors of the supply chain or global projects (new production line, new product, etc.).	AI plays an important role in decision-making and risk management in project management activities. The PM and the CM models are able to enact the whole range of activities (task) that should be developed by ensuring the good execution of the project.	[43]

**Table 3:** Advantages and disadvantages of the BPMN-PM unsupervised and supervised algorithms.

BPMN Approach	Advantages	Disadvantages
Integrating Unsupervised Algorithm	Useful for a large number of attributes (unknown “weight” of each attribute to be analyzed); Possibility of the integration of different data sources including an open dataset; Facilitates the implementation of complex PM models; Construction of risk maps; Does not require significant data- pre-processing (data filtering or data cleaning).	Can be adopted only for data clustering and not for forecasting; It is not easy to decide the best number of clusters to analyze (a low number of clusters facilitates the reading of the results but does not provide details about risk levels and risks classifications).
Integrating Supervised Algorithm	Facilitates the addressing of the analysis to a specific key variable; Can be adopted for data classification and for forecasting; Construction of risk maps; Can set more hyperparameters optimizing the analysis to be performed; Can be used for augmented data to improve the model when using a dataset with few records.	Requires a big dataset to optimize the AI training model; Requires data pre-processing; Wrong data in the training dataset could drastically increase the error probability; More suitable for a low number of variables; Could add redundancy in the analysis, thus increasing the estimation error.

**Figure A1:**
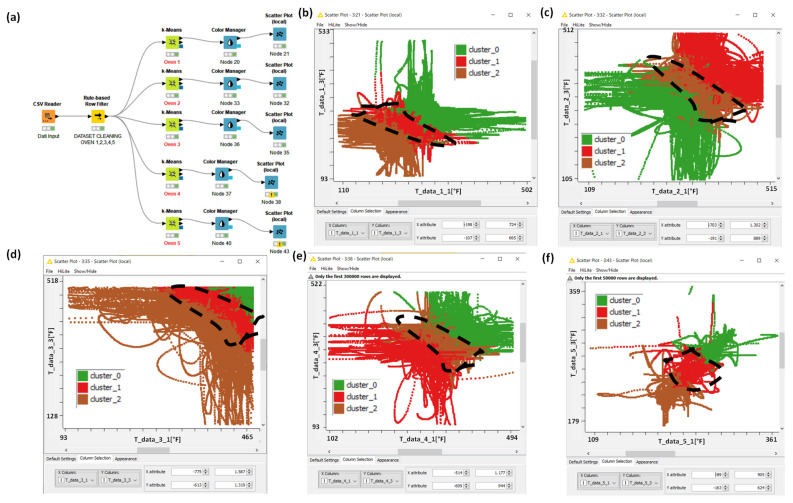
(**a**) KNIME workflow implementing unsupervised k-Means algorithm for oven data clustering. Couple of temperature variables and related data clustering (k = 3) for oven 1 (**b**), oven 2 (**c**), oven 3 (**d**), oven 4 (**e**), and oven 5 (**f**). The dashed black lines enclose the stable regions.

**Figure A2:**
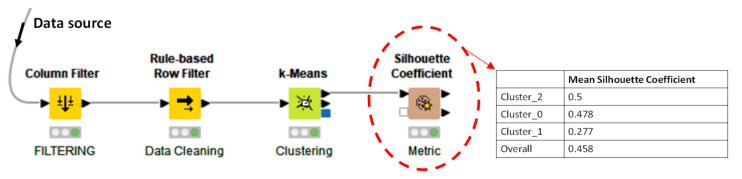
KNIME workflow implementing Silhouette Coefficient estimating clustering performance.

**Figure A3:**
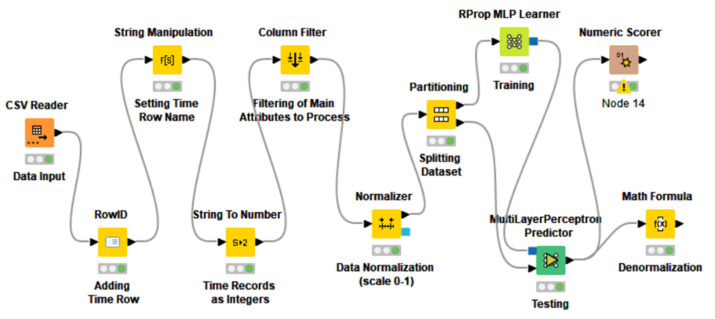
KNIME workflow of implementing the ANN-MLP algorithm in predicting energy power.

## Appendix A. Roasting Production Process and DSS Implementing an AI Unsupervised Model

The KNIME workflow of Figure A1a is implemented to perform the k-Means clustering analysis (k = 3) defining the risk maps illustrated in Figure A1b–f. For each oven, we have identified a stable cluster (no risk region), indicated by the black dashed line. Data pre-processing is executed to clean the dataset of anomalous temperature values (only values <500 °F and >100 °F are considered for the k-Means data processing); the cleaning process is performed by means of a structured logic condition (AND logic applied simultaneously for all five ovens). The increase in the cluster number k could help to quantitatively define the boundary conditions for the moderate alerting levels.

The metric adopted for the estimation of clustering performance is the Silhouette Coefficient [44]. Figure A2 shows the KNIME workflow implementing the “Silhouette Coefficient” and the calculated mean Silhouette Coefficient (SC) for a specific oven and a cleaned dataset. The method estimates the expression:

SC = (*distance2* − *distance1*)/*max*(*distance1*,*distance2*) (A1)

where *distance1* is the mean intra-cluster distance and *distance2* the mean inter-cluster distance to the closest cluster. The algorithm calculates the mean overall individual and total Silhouette Coefficients with a score ranging from −1.0 (very low score) to 1.0 (very high score). According to the analyzed case, the choice of k = 3 is a good compromise between the simplicity of analysis and the performance of the clustering algorithm (the total overall coefficient of 0.458 is an average–high value).

## Appendix B. AI Supervised Algorithm Predicting Energy Power

The energy power prediction is performed by the KNIME workflow of Figure A3. The input data [25] are preprocessed to be used by the ANN-MLP algorithm (see RProp MLP Learner and MultiLayerPerceptron blocks). The data pre-processing is performed by defining the time variable and by selecting the other variables to process (PE as label variable, and T, AP, RH and V as further variables to process). Good algorithm performance is achieved (a Means Absolute Error (MAE) of 0.045 is observed). The ANN-MLP network is characterized by the following hyperparameters: maximum number of iterations equal to 1000, one hidden layer having 10 neurons, a training model constructed from 70% of the dataset, and a testing model constructed from the remaining 30% of the dataset (last records sorted in time).

In Table A1 are shown the statics regarding the ANN-MLP algorithm’s performance.

**Table A1:** Parameters of ANN-MLP performance.

Parameter	Value
R^2^	0.935
Mean Absolute Error (MAE)	0.045
Mean Squared Error (MSE)	0.003
Root Mean Squared Error (RMSE)	0.058
Mean Signed Difference (MSD)	0.002

## References

[1] Massaro A. (2021). Electronic in Advanced Research Industry: From Industry 4.0 to Industry 5.0 Advances.

[2] Drakoulogkonas P., Apostolou D. (2021). On the Selection of Process Mining Tools. Electronics.

[3] Lecture Notes in Business Information Processing. https://www.springer.com/series/7911.

[4] Brzychczy E., Gackowiec P., Liebetrau M. (2020). Data Analytic Approaches for Mining Process Improvement—Machinery Utilization Use Case. Resources.

[5] Vladareanu L. (2020). Advanced Intelligent Control through Versatile Intelligent Portable Platforms. Sensors.

[6] Karthik T.S., Kamala B. (2021). Cloud Based AI Approach for Predictive Maintenance and Failure Prevention. J. Phys. Conf. Ser..

[7] Massaro A., Manfredonia I., Galiano A., Pellicani L., Birardi V. (2019). Sensing and Quality Monitoring Facilities Designed for Pasta Industry Including Traceability, Image Vision and Predictive Maintenance. Proceedings of the 2019 II Workshop on Metrology for Industry 4.0 and IoT (MetroInd4.0&IoT).

[8] Mehdiyev N., Fettke P. (2021). Explainable Artificial Intelligence for Process Mining: A General Overview and Application of a Novel Local Explanation Approach for Predictive Process Monitoring. Studies in Computational Intelligence.

[9] Kim K., Kim B. (2022). Decision-Making Model for Reinforcing Digital Transformation Strategies Based on Artificial Intelligence Technology. Information.

[10] Pilipczuk O. (2021). Transformation of the Business Process Manager Profession in Poland: The Impact of Digital Technologies. Sustainability.

[11] Bellantuono N., Nuzzi A., Pontrandolfo P., Scozzi B. (2021). Digital Transformation Models for the I4.0 Transition: Lessons from the Change Management Literature. Sustainability.

[12] Fetais A., Abdella G.M., Al-Khalifa K.N., Hamouda A.M. (2022). Business Process Re-Engineering: A Literature Review-Based Analysis of Implementation Measures. Information.

[13] Massaro A., Galiano A. (2020). Re-Engineering Process in a Food Factory: An Overview of Technologies and Approaches for the Design of Pasta Production Processes. Prod. Manuf. Res..

[14] Massaro A., Contuzzi N., Galiano A., Manfredonia I., Xhahysa B. (2019). A Preliminar Research Industry Project: A Case of Study Defining Requirements for Knowledge Base Gain and Technological Upgrade in Industry Working in Train Parts Processing and Testing. Proceedings of the 2019 II Workshop on Metrology for Industry 4.0 and IoT (MetroInd4.0&IoT).

[15] Liagkou V., Stylios C., Pappa L., Petunin A. (2021). Challenges and Opportunities in Industry 4.0 for Mechatronics, Artificial Intelligence and Cybernetics. Electronics.

[16] Fernandes J., Reis J., Melão N., Teixeira L., Amorim M. (2021). The Role of Industry 4.0 and BPMN in the Arise of Condition-Based and Predictive Maintenance: A Case Study in the Automotive Industry. Appl. Sci..

[17] Zareen S., Akram A., Ahmad Khan S. (2020). Security Requirements Engineering Framework with BPMN 2.0.2 Extension Model for Development of Information Systems. Appl. Sci..

[18] Massaro A. (2021). Information Technology Infrastructures Supporting Industry 5.0 Facilities. Electronics in Advanced Research Industries.

[19] Releases Notes for 20.3.0. https://github.com/jgraph/drawio-desktop/releases.

[20] Berthold M.R., Cebron N., Dill F., Gabriel T.R., Kötter T., Meinl T., Ohl P., Sieb C., Thiel K., Wiswedel B. (2008). KNIME: The Konstanz Information Miner. Data Analysis, Machine Learning and Applications.

[21] KNIME. https://www.knime.com/.

[22] Massaro A., Cosoli G., Leogrande A., Magaletti N. (2022). Predictive Maintenance and Engineered Processes in Mechatronic Industry: An Italian Case Study. Int. J. Artific. Appl..

[23] Production Quality. https://www.kaggle.com/datasets/podsyp/production-quality.

[24] Ahmed M., Seraj R., Islam S.M.S. (2020). The *k-means* Algorithm: A Comprehensive Survey and Performance Evaluation. Electronics.

[25] Combined Cycle Power Plant. https://www.kaggle.com/datasets/shivendraverma/combined-cycle-power-plant.

[26] Taleb I., Guerard G., Fauberteau F., Nguyen N. (2022). A Flexible Deep Learning Method for Energy Forecasting. Energies.

[27] Saloko S., Sulastri Y., Murad, Rinjani M.A. (2019). The Effects of Temperature and Roasting Time on the Quality of Ground Robusta Coffee (Coffea Rabusta) Using Gene Café Roaster. Proceedings of the 2nd International Conference on Bioscience, Biotechnology, and Biometrics 2019.

[28] Catelani T.A., Páscoa R.N.M.J., Santos J.R., Pezza L., Pezza H.R., Lima J.L.F.C., Lopes J.A. (2017). A Non-Invasive Real-Time Methodology for the Quantification of Antioxidant Properties in Coffee during the Roasting Process Based on near-Infrared Spectroscopy. Food Bioprocess Technol..

[29] Schiuma G., Lerro A., Sanitate D. (2008). The Intellectual Capital Dimensions of Ducati’s Turnaround: Exploring Knowledge Assets Grounding a Change Management Program. Int. J. Innov. Manag..

[30] Massaro A. (2022). “Energetic” KPI in Logistics: Complex System Theory and Multi-Level Modeling. Zenodo. https://zenodo.org/record/6137729#.Y1lLGXZBxPY.

[31] Vujanovic D., Mijailovic R., Momcilovic V., Papic V. (2010). Energy Efficiency as a Criterion in the Vehicle Fleet Management Process. Therm. Sci..

[32] Massaro A., Starace G. (2022). Advanced and Complex Energy Systems Monitoring and Control: A Review on Available Technologies and Their Application Criteria. Sensors.

[33] Massaro A., Panarese A., Gargaro M., Colonna A., Galiano A. (2021). A Case Study of Innovation in the Implementation of a DSS System for Intelligent Insurance Hub Services. Comput. Sci. Inf. Technol..

[34] Massaro A. (2021). Rapid Prototyping. Electronics in Advanced Research Industries.

[35] Alnamrouti A., Rjoub H., Ozgit H. (2022). Do Strategic Human Resources and Artificial Intelligence Help to Make Organisations More Sustainable? Evidence from Non-Governmental Organisations. Sustainability.

[36] Achchab S., Temsamani Y.K. Artificial Intelligence Use in Human Resources Management: Strategy and Operation’s Impact. Proceedings of the 2021 IEEE 2nd International Conference on Pattern Recognition and Machine Learning (PRML).

[37] Tewari I., Pant M. Artificial Intelligence Reshaping Human Resource Management: A Review. Proceedings of the 2020 IEEE International Conference on Advent Trends in Multidisciplinary Research and Innovation (ICATMRI).

[38] Massaro A. (2021). Electronic and Reverse Engineering. Electronics in Advanced Research Industries.

[39] Chouchene A., Carvalho A., Lima T.M., Charrua-Santos F., Osorio G.J., Barhoumi W. (2020). Artificial Intelligence for Product Quality Inspection toward Smart Industries: Quality Control of Vehicle Non-Conformities. Proceedings of the 2020 9th International Conference on Industrial Technology and Management (ICITM).

[40] Papageorgiou E.I., Theodosiou T., Margetis G., Dimitriou N., Charalampous P., Tzovaras D., Samakovlis I. (2021). Short Survey of Artificial Intelligent Technologies for Defect Detection in Manufacturing. Proceedings of the 2021 12th International Conference on Information, Intelligence, Systems & Applications (IISA).

[41] Damacharla P., Rao A., Ringenberg J., Javaid A.Y. (2021). TLU-Net: A Deep Learning Approach for Automatic Steel Surface Defect Detection. Proceedings of the 2021 International Conference on Applied Artificial Intelligence (ICAPAI).

[42] Massaro A., Manfredonia I., Galiano A., Xhahysa B. (2019). Advanced Process Defect Monitoring Model and Prediction Improvement by Artificial Neural Network in Kitchen Manufacturing Industry: A Case of Study. Proceedings of the 2019 II Workshop on Metrology for Industry 4.0 and IoT (MetroInd4.0&IoT).

[43] Smith C.J., Wong A.T.C. (2022). Advancements in Artificial Intelligence-Based Decision Support Systems for Improving Construction Project Sustainability: A Systematic Literature Review. Informatics.

[44] Aranganayagi S., Thangavel K. (2007). Clustering Categorical Data Using Silhouette Coefficient as a Relocating Measure. Proceedings of the International Conference on Computational Intelligence and Multimedia Applications (ICCIMA 2007).

